# Supplementary material for: IgE-mediated mast cell activation promotes inflammation and cartilage destruction in osteoarthritis
Source: eLife. 2019 May 14;8:e39905. doi: 10.7554/eLife.39905 (PMC6516833; doi:10.7554/eLife.39905)
Supplement: Transparent reporting form [file elife-39905-transrepform.pdf]

eLIFE
1st Floor
24 Hills Road
Cambridge CB2 1JP, UK
P 01223 855340
W elifesciences.org
T @elife

eLife's transparent reporting form

We encourage authors to provide detailed information *within their submission* to facilitate the interpretation and replication of experiments. Authors can upload supporting documentation to indicate the use of appropriate reporting guidelines for health-related research (see EQUATOR Network), life science research (see the BioSharing Information Resource), or the ARRIVE guidelines for reporting work involving animal research. Where applicable, authors should refer to any relevant reporting standards documents in this form.

If you have any questions, please consult our Journal Policies and/or contact us: editorial@elifesciences.org.

Sample-size estimation

- You should state whether an appropriate sample size was computed when the study was being designed
- You should state the statistical method of sample size computation and any required assumptions
- If no explicit power analysis was used, you should describe how you decided what sample (replicate) size (number) to use

Please outline where this information can be found within the submission (e.g., sections or figure legends), or explain why this information doesn't apply to your submission:

Determination of sample sizes for analysis of human synovial fluid samples are described in the "Measurement of active tryptase in synovial fluids" sub-section of the Materials and Methods. Sample sizes are provided in the legend for Figure 1.

Determination of sample sizes for analysis of cartilage degradation, osteophyte formation and synovitis following destabilization of the medial meniscus in mice were determined using power calculations as is described in the "Surgical induction of osteoarthritis in mice" sub-section of the Materials and Methods. Sample sizes used in each experiment are provided in the legends of Figures 2, 3, 4 and 5.

qPCR analysis of inflammatory/degradative enzyme expression was performed on mouse joint tissue samples derived from other experiments presented in the current manuscript, in which sample sizes were determined by power calculations as described above.

Replicates

- You should report how often each experiment was performed
- You should include a definition of biological versus technical replication
- The data obtained should be provided and sufficient information should be provided to indicate the number of independent biological and/or technical replicates
- If you encountered any outliers, you should describe how these were handled
- Criteria for exclusion/inclusion of data should be clearly stated

- High-throughput sequence data should be uploaded before submission, with a private link for reviewers provided (these are available from both GEO and ArrayExpress)

Please outline where this information can be found within the submission (e.g., sections or figure legends), or explain why this information doesn't apply to your submission:

Biological and technical replicate numbers for the analysis of human synovial fluid samples are provided in the legend for Figure 1 and in the "Transmission electron microscopy (TEM) analysis of human synovium" sub-section of the Materials and Methods.

Biological and technical replicate numbers for analysis of cartilage degradation, osteophyte formation and synovitis following destabilization of the medial meniscus in mice are provided in the legends of Figures 2, 3, 4 and 5.

Biological and technical replicate numbers for qPCR analysis of inflammatory and degradative enzyme expression are provided in the legend for Figure 3.

Statistical reporting

- Statistical analysis methods should be described and justified
- Raw data should be presented in figures whenever informative to do so (typically when N per group is less than 10)
- For each experiment, you should identify the statistical tests used, exact values of N, definitions of center, methods of multiple test correction, and dispersion and precision measures (e.g., mean, median, SD, SEM, confidence intervals; and, for the major substantive results, a measure of effect size (e.g., Pearson's r, Cohen's d)
- Report exact p-values wherever possible alongside the summary statistics and 95% confidence intervals. These should be reported for all key questions and not only when the p-value is less than 0.05.

Please outline where this information can be found within the submission (e.g., sections or figure legends), or explain why this information doesn't apply to your submission:

Methods of statistical analysis of tryptase levels and mast cell degranulation in human synovial fluid samples are provided in the legend for Figure 1 and "Statistics" sub-section of the Materials and Methods.

Methods of statistical analysis of cartilage degradation, osteophyte formation and synovitis following destabilization of the medial meniscus in mice are provided in the legends of Figures 2, 3, 4 and 5 and "Statistics" sub-section of the Materials and Methods.

Methods of statistical analysis of expression of inflammatory and degradative mediators in cartilage and synovial fibroblasts treated with tryptase are provided in the legend of Figure 3 and "Statistics" sub-section of the Materials and Methods.

(For large datasets, or papers with a very large number of statistical tests, you may upload a single table file with tests, Ns, etc., with reference to sections in the manuscript.)

Group allocation

- Indicate how samples were allocated into experimental groups (in the case of clinical studies, please specify allocation to treatment method); if randomization was used, please also state if restricted randomization was applied
- Indicate if masking was used during group allocation, data collection and/or data analysis

Please outline where this information can be found within the submission (e.g., sections or figure legends), or explain why this information doesn't apply to your submission:

Description of masking for assessment of cartilage damage, synovial thickening and osteophyte formation for destabilization of medial meniscus experiments is described in the "Histologic assessment of osteoarthritic development in mice" sub-section of the Materials and Methods.

Masking was not applicable for other experiments because of the use of unbiased data collection methods.

Additional data files ("source data")

- We encourage you to upload relevant additional data files, such as numerical data that are represented as a graph in a figure, or as a summary table
- Where provided, these should be in the most useful format, and they can be uploaded as "Source data" files linked to a main figure or table
- Include model definition files including the full list of parameters used
- Include code used for data analysis (e.g., R, MatLab)
- Avoid stating that data files are "available upon request"

Please indicate the figures or tables for which source data files have been provided:

No additional data or source data is available for this manuscript.
